# Genetic Diversity and Population Structure of *Macrobrachium nipponense* Populations in the Saline–Alkaline Regions of China

**DOI:** 10.3390/ani15020158

**Published:** 2025-01-09

**Authors:** Romaric Iko, Zijian Gao, Sufei Jiang, Yiwei Xiong, Wenyi Zhang, Hui Qiao, Shubo Jin, Hongtuo Fu

**Affiliations:** 1 Wuxi Fisheries College, Nanjing Agricultural University, Wuxi 214081, China; romariciko@gmail.com (R.I.); gaozijiangenomics@163.com (Z.G.); 2 Key Laboratory of Freshwater Fisheries and Germplasm Resources Utilization, Ministry of Agriculture, Freshwater Fisheries Research Center, Chinese Academy of Fishery Sciences, Wuxi 214081, China; jiangsf@ffrc.cn (S.J.); xiongyw@ffrc.cn (Y.X.); zhangwy@ffrc.cn (W.Z.); qiaoh@ffrc.cn (H.Q.)

**Keywords:** genetic diversity, population structure, *Macrobrachium nipponense*, D-loop, saline alkaline

## Abstract

The oriental river prawn *Macrobrachium nipponense* is of great economic importance in China. However, its culture in the saline–alkaline regions is limited. This study used D-loop region sequences to analyze the genetic diversity and population structure across the saline–alkaline regions of China. A total of 264 individuals were successfully sequenced from nine sites, including Daqing in Heilongjiang Province (HLJ), Songyuan in Jilin Province (JL), Ulanhot in the east of inner Mongolia (NMG), Jingtai in Gansu Province (GS), Dongying in Shandong Province (SD), Dongtai in Jiangsu Province (JS), Nanchang in Jiangxi province (JX), Tianjin in mainland China (TJ), and Yinchuan in Ningxia Province (NX). In addition, 89 haplotypes were defined. The haplotype diversity (*h*) and nucleotide diversity π showed remarkable genetic diversity in the JS, NX, JL, and TJ sites. It was found that the genetic variation within sites was relatively greater. The genetic distance (D) and the pairwise genetic differentiation index (F_ST_) revealed that SD and GS are closely related. The F_ST_ values among the nine sites were significant except for the one between SD and HLJ (*p*-value > 0.05). The cluster analyses and the phylogenetic tree identified two main groups. There is an association among sites by geographic location, and the JS site shows higher diversity. The results of this study provide basic information for the protection and development of *M. nipponense* resources in the saline–alkaline regions of China.

## Simple Summary

This study assesses the genetic diversity and population structure of the oriental river prawn *Macrobrachium nipponense* across the saline–alkaline regions of China using mitochondrial D-loop region sequences. Genetic differentiation within the nine sites is high, and four sites showed higher genetic diversity. Population genetic structure analyses grouped the sites into two different clusters, and there is a relationship among sites by geographic location. The genetic distance D and pairwise genetic differentiation Fst illustrated a moderate level of gene flow among the sites, two of which exhibited strong genetic similarity. This finding can guide a potential genetic breeding program to produce a new variety with strong ability to resist the saline–alkaline treatment, maintaining the sustainable development of *M. nipponense* in the saline–alkaline regions of China.

## 1. Introduction

The culture of aquatic animals in the saline–alkaline regions is beneficial not only for the sustainable development of the aquaculture industry but also for the rational utilization of water resources [1,2]. In rural and arid areas, where there is an acute shortage of fresh water, the necessity and importance of this optimization cannot be overstated [3]. The global area of saline–alkaline land is currently around 10 × 10^7^ Km^2^, corresponding to about 25% of the Earth’s land area [4,5]. In China, there are approximately 99.13 million hectares of saline–alkaline land, which are primarily located in the northeast, northwest, and coastal areas [6]. Therefore, inland saline–alkaline water aquaculture has become a crucial strategy to reduce freshwater consumption and promote aquaculture development in saline-affected regions.

Crustacean species often have a lower alkalinity tolerance than fish species [7]. For example, *Gymnocypris przewalskii* have a safe alkali value of 64 mmol/L [8]. *Tribolodon brandti* has *LC*_50_ values of 98.79 mmol/L, 89.31 mmol/L, 79.34 mmol/L, 78.45 mmol/L, and 68.44 mmol/L, respectively, at 12 h, 24 h, 48 h, 72 h, and 96 h with an optimal value of 18.79 mmol/L [9]. *Palaemon przewalskii* can only tolerate 3.5 mmol/L [10], and the suitable value for *Penaeus vannamei* is 2.90 mmol/L [11] for optimal growth.

*Macrobrachium nipponense* (de Haan, 1849), commonly known as the oriental river prawn, is a species of significant ecological and economic importance in China [12]. The main region for *M. nipponense* culture is in the southern part, including Jiangsu Province, Anhui Province, Zhejiang Province, and Jiangxi Province, producing huge economic benefits [13]. The production in the northern part of China is limited. *M. nipponense* lacks sufficient alkali tolerance to thrive in water environments with high alkali concentrations. A previous study has identified that the alkaline *LC*_50_ at 12 h, 24 h, 48 h, 72 h, and 96 h in juvenile prawns of “Taihu No. 2” (a new variety of *M. nipponense*) were 27.66 mmol/L, 26.94 mmol/L, 22.51 mmol/L, 15.00 mmol/L, and 14.42 mmol/L, respectively, with the safety value of 4.71 mmol/L [14]. For the sustainable development of the *M. nipponense* industry and with the scarcity of freshwater, it is important to identify potential varieties of *M. nipponense* with excellent genetic diversity for the species’ sustainability and food security. The analysis of the *M. nipponense* population in China has increased in recent years [15,16,17]. However, most of these genetic structure analysis researchers were focused on the genetic diversity of *M. nipponense* populations in major producing areas or important water areas [18,19,20,21]. A study of the genetic diversity and population structure of *M. nipponense* in the saline alkaline area has not yet been conducted. The genetic diversity and population structure of *M. nipponense* cultured in the saline–alkaline region of China will provide a basis to establish a foundation for the protection and utilization of *M. nipponense* germplasm resources in those regions.

Mitochondrial DNA (mtDNA) is widely used in studies on population genetics and molecular systematics because it is strongly conserved across animals, has very few duplications, evolves rapidly, and has very short intergenic regions [22,23]. Because mutations accumulate most rapidly in the D-loop gene, this region of mtDNA is preferred for population and phylogenetic studies among closely related species, including crustaceans [24,25]. Mitochondrial DNA (mtDNA) based on the D-loop gene has been widely used for genetic analysis from multiple populations in *M. nipponense* [15,17]. A study of the genetic structure of twenty-two populations of *M. nipponense* across different regions of China shows a steady increase among the population with significant diversity in the southern part of the country [17]. The genetic diversity of eight populations of *M. nipponense* in Yixing city with Taihu Lake, Yangtze River, and the main local artificial strain ‘Taihu No. 2’ reveals that the genetic diversity of *M. nipponense* in Yixing was in excellent condition [16]. This study is also based on D-loop region sequences to evaluate the genetic diversity and population structure of *M. nipponense* across the saline–alkaline region of China.

## 2. Materials and Methods

### 2.1. Ethics Statement

No endangered or protected species were involved in this study. For all experimental protocols, the Animal Care and Use Ethics Committee of the Freshwater Fisheries Research Center (Wuxi, China) approved all the methods in December 2023 (Authorization No. 20231211003).

### 2.2. Sample Collection

**Figure 1:**
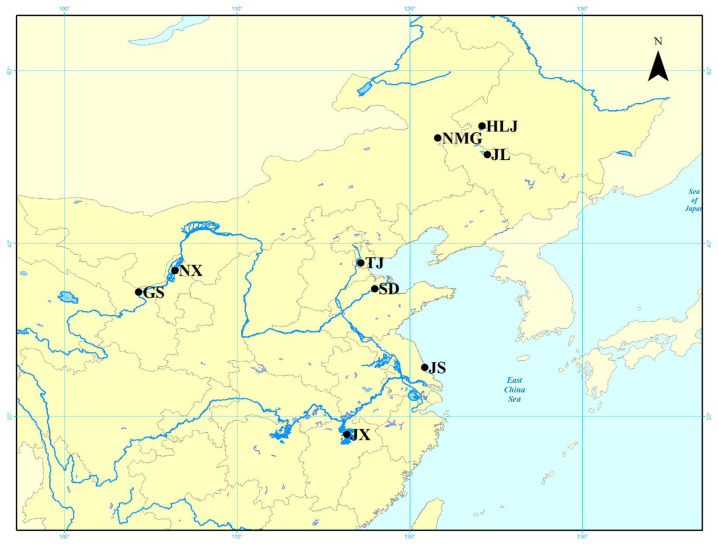
The sampling location of *M. nipponense* in the saline–alkaline regions of China.

Samples of wild *M. nipponense* were collected from the saline–alkaline water area in China, as shown in Figure 1. The different sampling sites were as follows: Heilongjiang Province (HLJ) in Daqing city, Jilin Province (JL) in Songyuan city, the east of Inner Mongolia (NMG) in Ulanhot city, Gansu Province (GS) in Jingtai city, Shandong Province (SD) in Dongying city, Jiangsu Province (JS) in Dongtai city, Jiangxi province (JX) in Nanchang city, mainland China in Tianjin (TJ), and Ningxia Province (NX) in Yinchuan city. Table 1 gives details on the geographical coordinates of each location, the salinity and alkalinity of each sampling location, and individuals used for mitochondrial marker analysis. A total of 315 individuals from nine sites were collected. Muscle tissue samples were stored in 95% ethanol.

### 2.3. DNA Preparation, PCR Amplification, and Sequencing

About 50 mg of muscle was sampled from each individual. Genomic DNA was extracted from 35 individuals of each site using the phenol–chloroform method [26]. PCR was conducted using 50 µL reaction mixtures according to the method of Jiang et al. [27]. The purity and concentration of the DNA were detected using the nanodrop spectrophotometer. The DNA concentration of each sample was adjusted to about 20 ng/µL and kept under −20 °C until ready to use. Primers for the D-loop gene of *M. nipponense* (Accession number: MZ614458) were designed as F: 5′-TTTACTCCCAGTCTAACC-3′, R: 5′-TTCATTATTCGCCCTATC-3′. Prime-blast was used to verify the specificity of primers based on the nt database according to the mitochondrial DNA of macrobrachium species. Self-complementarity values were 3.00 and 3.00. Self 3′ complementarity values were 0.00 and 0.00. Then, the PCR products were detected using 1% agarose gel electrophoresis, and the bidirectional sequencing was carried out with an automated sequencer (Invitrogen Biotechnology Co., Ltd., Carlsbad, CA, USA). The sequencer was based on the first Generation DNA-sequencing method.

### 2.4. Data Analysis

To ensure accuracy, all DNA fragments were sequenced in two directions, and the assembled sequences were manually checked to prevent the base or sequencing error ambiguity. After the completion of the splicing, all sequences were used for homologous alignment and length determination with BioEdit version 7.0.9 software [28]. The nucleotide configurations, nucleotide diversity (π), and haplotype diversity (*h*) were calculated with DnaSP 6.0 (http://www.ub.edu/dnasp/ (accessed on 24 October 2024)). The analysis of molecular variance model (AMOVA) was used to estimate genetic variation following 1000 permutations in ARLEQUIN 3.5 [29]. Genetic distances within and among the sites were estimated with MEGA 7 [30]. Pairwise genetic differentiation (F_ST_) was calculated with 10,000 permutations in ARLEQUIN 3.5, and the false discovery rate (FDR) was implemented using the method of Benjamini and Hochberg [31]. Bootstrap values were based on 1000 rapid bootstrap replicates. The haplotype network was constructed using the median-joining method in POPART 1.7 [32]. During the alignment, the sequences with identity or cover rate below 90 were deleted, resulting in a smaller sample size for population structure analysis than in collection. In this process, sequences with large gaps and missing data are filtered out. These sequences were not further addressed for haplotype network construction because some of the small gaps might have been caused by insertion or deletion.

A phylogenetic tree was built using the neighbor-joining method based on the genetic distance by MEGA 7 [33]. The principal components analysis (PCA) was performed using the R package adegenet (http://cran.r-project.org/mirrors.html (accessed on 24 October 2024)). The population structure was investigated with a Bayesian model-based cluster procedure using Structure version 2.3.4 [34]. This method uses Markov chain Monte Carlo (MCMC) methods to estimate allele frequencies and to identify the optimum number of clusters (K) that suit the data. The following settings were used: a burn-in period of 10,000 iterations followed by 100,000 recorded iterations for K = 1 to K = 6 clusters. Structure Harvester online was used to analyze the maximum value of ΔK associated with each K value according to Evanno’s approach [35,36].

## 3. Results

### 3.1. D-Loop Sequences Analysis

In total, 264 D-loop sequences were obtained from different saline alkaline regions. A total of 231 variable sites were identified, accounting for 23% of the total available sites. The JS region had the most polymorphic sites (145), whereas the least (58) was found in NMG (Table 2).

### 3.2. Haplotype Distribution

**Figure 2:**
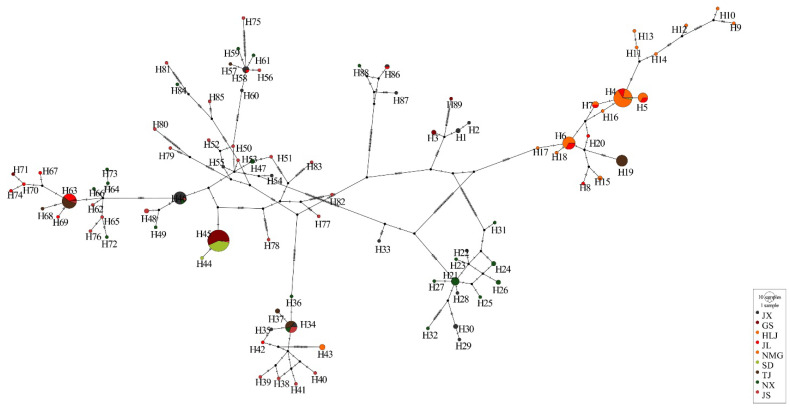
The median-joining network based on haplotype frequencies of *M. nipponense*. Each circle represents a haplotype, and the size of the circle is proportional to the number of individuals in the sampling site. Each line in the network represents a single mutational change, and the branches are scaled to the number of polymorphic sites between each haplotype. Each black circle represents one missing haplotype.

A total of 89 haplotypes were defined from 264 individuals of *M. nipponense* sequenced from nine saline alkaline regions (Table S1). Among these, nine shared haplotypes were detected. Hap4, Hap34 and Hap63 were the dominant haplotypes, and all were shared by four sampling sites. Three sites shared Hap45 and Hap46. Hap5, Hap6, Hap7, and Hap58 were common to two sites. The remaining haplotypes were unique to different sites. The highest number (26) of unique haplotypes were found in the JS region, while SD had the least unique (1) haplotype. The haplotype network (Figure 2) illustrated that haplotypes were linked from different regions; all ten shared haplotypes and the three dominants were clearly defined from others. The haplotype distribution of NX and JS were relatively scattered, while that of SD and NMG were concentrated.

### 3.3. Population Variation

Table 3 displays the analysis of molecular variance (AMOVA), showing details on the distribution of the genetic variation within and among the saline alkaline regions *M. nipponense*. According to the result, genetic differences among sites accounted for 36.81% of the genetic variation, whereas variance within sites accounted for 63.19%. Within the locations, the variance component was 26.29% and the sum of squares was 6705.13. The Fst value was high (Fst = 0.36810) and significant (*p*-value < 0.01), showing a high degree of genetic differentiation among the 09 *M nipponense* sampling sites.

### 3.4. Population Genetic Diversity

#### 3.4.1. Haplotype Diversity (*h*) and Nucleotide Diversity (π)

The average haplotype diversity (*h*) was 0.94. The highest *h* (1) was found in the JS site, and the lowest (0.879) was observed in the JX site. All the sites identified had an *h* value of >0.9 except that of the JX site. With regard to nucleotide diversity, the overall was 0.032 with values ranging from 0.012 (SD) to 0.037 (NX). Five sites (NX, TJ, JS, JL, JX) showed π values above 0.03 and four sites (SD, NMG, GS, HLJ) have π values between 0.012 and 0.016 (Table 2).

#### 3.4.2. Pairwise Fst

The pairwise genetic differentiation index (F_ST_) values between these nine sites are shown in Table 4. The Fst values vary greatly from 0.000 (between SD and GS) to 0.812 (between SD and NMG), indicating a considerable degree of divergence between SD and NMG sites. All Fst values among the nine sites were significant except for the one between the SD and HLJ sites (*p*-value > 0.05).

#### 3.4.3. Genetic Distances

The genetic distances among the nine sites are shown in Table 5. The genetic distance ranged from 0.020 to 0.078. The highest (0.078) was observed between the JS and NMG sites, which was followed by the genetic distances between the NX and NMG sites and the JS and HLJ sites with the value of 0.071. The lowest (0.020) was observed among the SD and GS sites.

### 3.5. Phylogenetic and Population Structure

**Figure 3:**
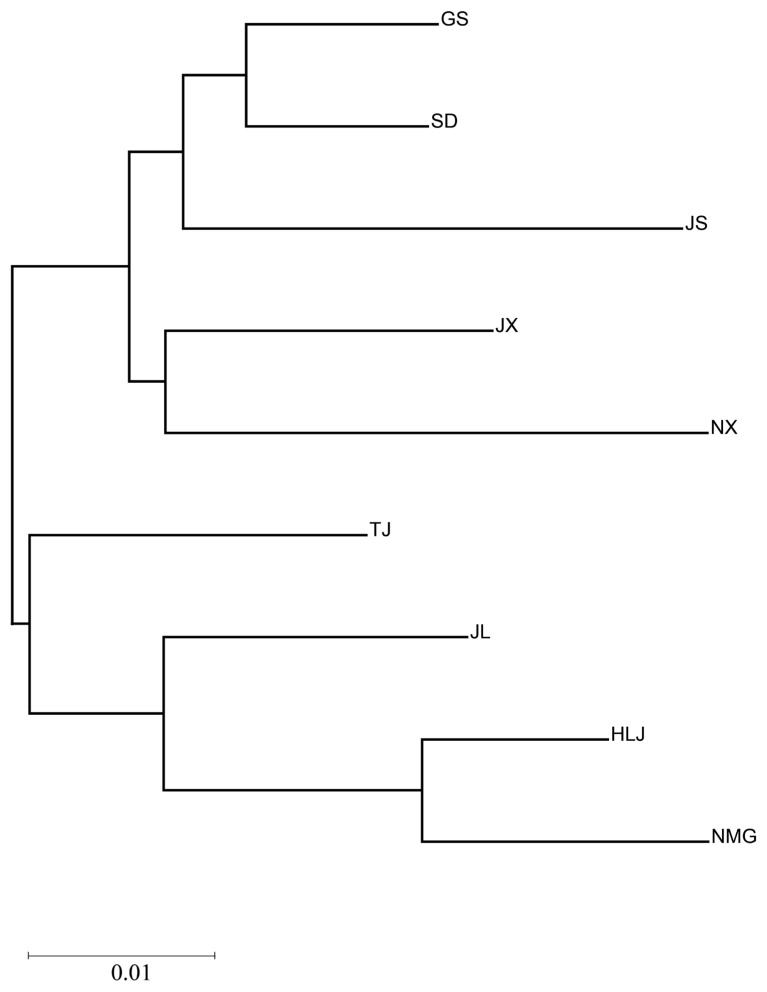
Phylogenetic tree based on genetic distances for 9 *M. nipponense* sampling sites.

The phylogenetic tree based on the genetic distance of the nine sites of *M. nipponense* was constructed (Figure 3). Two main groups were identified. The first group comprised all sites from northeast China (JL, HLJ, NMG) clustered into one branch with TJ. The second group contained the following sites: SD, JS, JX, GS, NX. In this group, SD and GS were clustered together and then clustered with JS. Similarly, JX and NX were clustered together.

**Figure 4:**
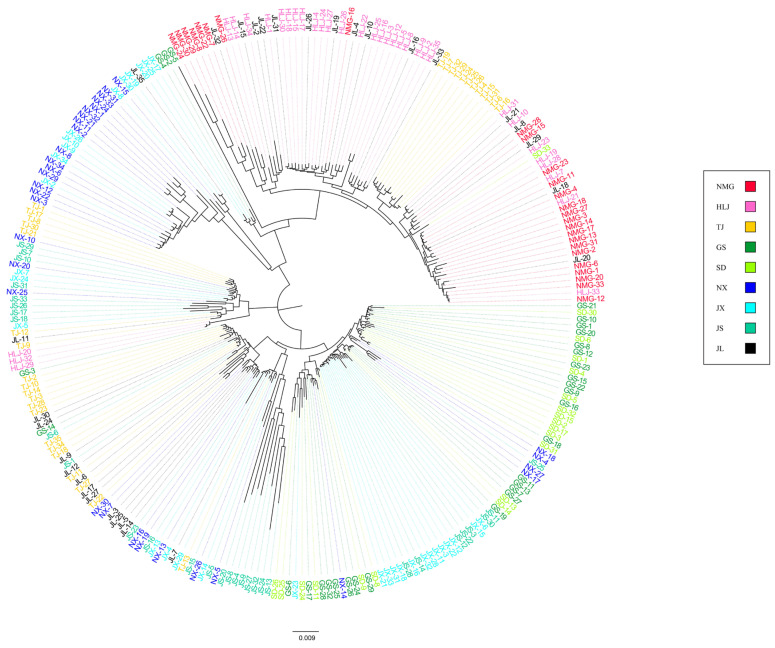
Neighbor-joining tree for 9 *M. nipponense* sites, each identical color representing individuals in a particular location.

The neighbor-joining tree among the sites shows that most genes of the *M. nipponense* from the northeast were clustered in one branch and closely related to one another like the remaining sites in the second branch (Figure 4).

**Figure 5:**
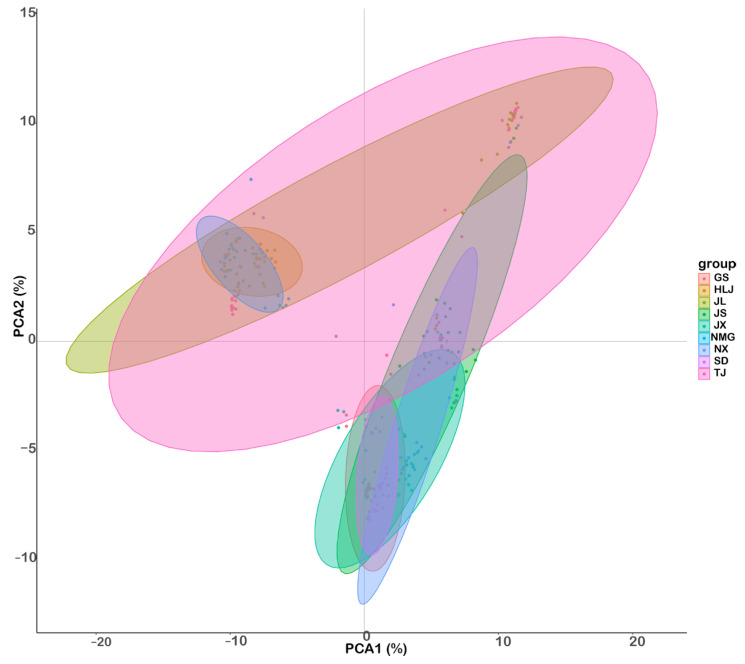
The principal components analysis (PCA) of 9 *M. nipponense* sites showing two main clustering. Each color represents a specific site.

The PCA analyses are widely used to determine the most important variables contributing to variation for easy visualization of the nine locations. The analysis corroborates the result obtained from the phylogenetic tree. There is a considerable cluster among the sites by geographic location. All the sites from the northeast clustered together and the remaining sites clustered separately. It is also illustrated that the TJ site was roughly scattered among the other sites (Figure 5).

**Figure 6:**
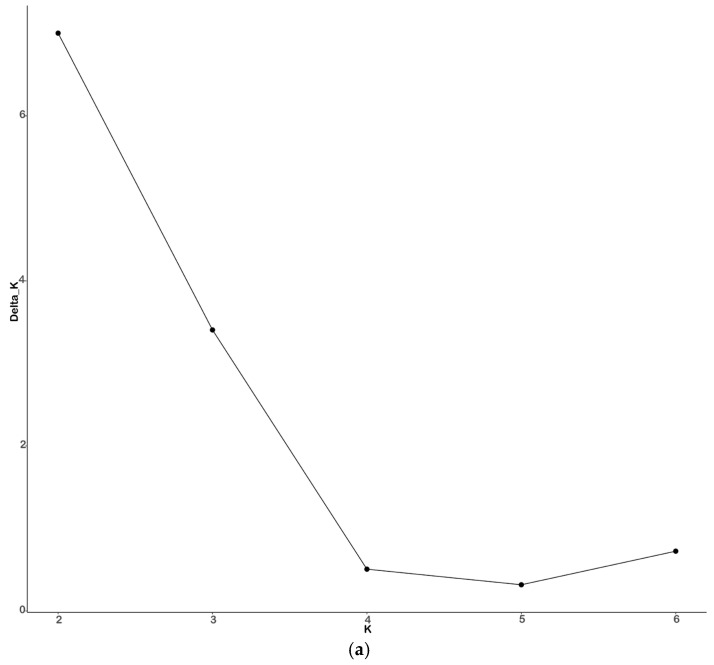

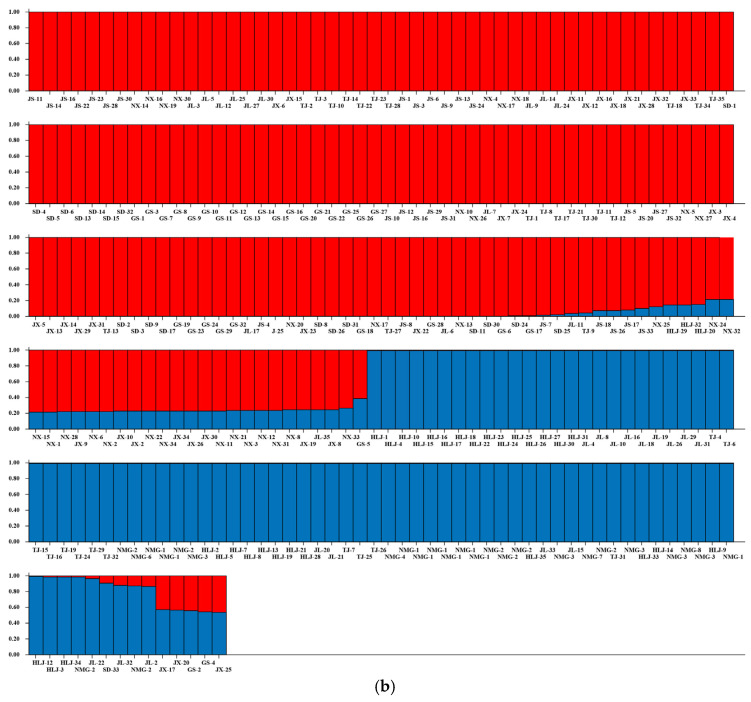
The results of structure analysis: (**a**) plot of delta K distribution of 9 *M. nipponense* sites; (**b**) bar plot of 9 *M. nipponense* sites with K = 2. Group 1 (blue bars) and Group 2 (red bars). The mixed color indicates individuals having ancestry from more than one site. The *x*-axis indicates the sampling site, and the *y*-axis shows the proportion of each cluster within individual genomes.

The results of STRUCTURE analysis of 264 *M. nipponense* in the alkaline regions of China showed that ΔK reached its peak at K = 2 (Figure 6a). The maximum value of ΔK at K = 2 was considered as the ideal number of groups.

## 4. Discussion

This study provides insight into the genetic diversity and population structure of *M. nipponense* specifically in the saline–alkaline region of China using D-loop region sequences with 264 individuals from nine saline alkaline regions. *M. nipponense* lacks sufficient tolerance to thrive in water environments with high alkali concentrations, which limits its production in those regions [7]. Due to the limited data available on the genetic diversity of the oriental river prawn in those regions, it is important to identify populations with strong genetic backgrounds to ensure the sustainable development of the *M. nipponense.*

Genetic diversity is the total amount of genetic variation between different populations within a species or among different individuals within a population, which provides the population the ability to cope with challenges such as parasites, pathogens, predators, and environmental changes [37,38,39]. There are many studies on the genetic diversity of *M. nipponense* in China using various molecular markers such as mitochondrial COI gene sequences [18], microsatellite markers [19], COI and 16SrRNA in mitochondrial DNA [19], and Amplified Fragment Length Polymorphism (AFLP) [40].

The detection of 231 variable sites emphasizes the important polymorphism in the D-loop region of *M. nipponense*; thus, it is considered an excellent marker for the oriental river prawn genetic diversity study, as suggested by previous research. This number is quite similar to 239 in another study of the genetic diversity of eight locations of *M. nipponense* [15]. A total of 348 variation sites were detected on 22 locations of the oriental river prawn [17]. However, 51 variable sites were detected in eight locations from the Three Gorges Reservoir in China using mitochondrial COI gene. The 16S rRNA and COI fragment, respectively, showed 78 and 42 variable sites among the oriental river prawn in East Asia [21]. This study considers the D-loop region of *M. nipponense* because of its high polymorphism compared to other mitochondrial regions.

Haplotype and nucleotide diversity are important indicators of genetic variation, and the larger the number, the greater the genetic variation in the group and the higher the genetic diversity [41]. Generally, all the studied regions exhibit higher haplotype diversity *h* varying from 0.879 to 1. The value of *h* in this study is higher than that of eight locations in China in a previous study (0.3684–0.981) [15] as well as for 11 locations (0.7438–0.9881) [16]. However, nucleotide diversity better reflects population genetic diversity than haplotype diversity [17,27]. It captures the average difference between nucleotides using SNPs and the genetic differences between them, providing a more comprehensive picture of variability [42]. The investigation shows that the nucleotide diversity and haplotype diversity are high in JS (*h =* 1, π = 0.032), NX (*h =* 0.987, π = 0.037), JL (*h =* 0.998, π = 0.034), and TJ (*h =* 0.972, π = 0.032). This suggests a healthy genetic diversity, likely a large effective size, and a moderate level of genetic variation at the nucleotide level in these regions. The SD, NMG, GS, and HLJ sites showed relatively lower nucleotide diversity (varies from 0.012 to 0.016), suggesting a higher risk of inbreeding depression. This also supports that the population in the lower reaches of Yangtze River in Jiangsu Province is an important germplasm resource as reported previously [40]. In addition, nine shared haplotypes were identified. The NX and JS sites exhibit a high level of unique haplotypes with 19 and 22 unique haplotypes, respectively. This also supports that they have rich genetic variation. Aside from the haplotypes of SD and NMG, all the remaining converged into different branches, implying that they were linked.

The result of AMOVA analysis showed a significant differentiation of 63.19% variation among individuals within the nine sites; this is relative to the previous studies [16,17,18] showing that diversity is more influenced by differences among individuals rather than differences between regions. *M. nipponense* is small and has limited ability to move, which makes long-distance migration challenging [18]. The genetic differentiation of this species may be more related to its introduction in some water areas like Boston Lake in the late 1960s [15].

Examining the population structure and patterns of gene flow among species is essential for understanding evolution [43]. The pairwise genetic differentiation Fst quantifies the proportion of genetic variation that occurs between specific pairs of populations and its contribution to genetic differentiation. All the Fsts in this study were significantly different aside from that of SD and HLJ (*p* > 0.05). The analyses revealed that the GS and SD sites are genetically identical (Fst = 0), which highlights a high gene flow among the two sites. Similarly, they are highly differentiated from NMG (Fst = 0.745 and Fst = 0.812, respectively) and HLJ (Fst = 0.695 and Fst = 0.750, respectively). Generally, there is a moderate level of gene flow among all the sites. The pairwise Fst values were similar to those of eight locations in China [15] and noticeably higher than the genetic differentiation index of the populations from Yangtze Rivers and Yixing City in China [16].

On the other hand, the pairwise genetic distance (D) ranged from 0.020 to 0.078, revealing that there is a moderate evolutionary divergence among the nine regions. The lower genetic distance of 0.020 observed among GS and SD corroborates the analysis of genetic differentiation among them. This low genetic dissimilarity stipulates that SD is closely related to the GS site. There might be no barriers to gene exchange among them.

Furthermore, the relationships among species are crucial for understanding phylogenetic classification [44]. In this study, the phylogenetic tree, PCA, and the neighbor-joining tree displayed the same result. They help to understand the evolutionary history and the genetic organization of the studied regions but lack details to interpret external factors that contribute to them.

The different sites can be categorized into two distinct groups based on the data. The structural analysis also indicated two groups corresponding to the peak of ΔK. It might be considered that the geographical location of the site affects group clustering. The TJ site is genetically related to the northeast sites (HLJ, NMG, JL) and these sites were clustered together; the second group can be composed of the southern sites (NX, GS, JX, JS, and SD).

It can be noticed that there is no correlation between the nucleotide diversity and the salinity and alkalinity concentration of the studied regions. The *p*-values were relatively greater (*p* > 0.05) for the Pearson correlation coefficient and Spearman correlation coefficient, which indicated that the salinity and alkalinity were not significant (Table S2). Therefore, salinity and alkalinity have limited influence on the genetic diversity.

## 5. Conclusions

The D-loop region sequences of nine sites of *M. nipponense* in the saline–alkaline regions of China reveal that four sites have outstanding genetic diversity, including JS, NX, JL, and TJ. Jiangsu Province (JS) in Dongtai can be considered as a potential candidate for genetic breeding. The SD and GS sites are more related than the remaining sites. There is an important level of association among the sites by geographic location. The saline–alkaline nature of the sampling environment has little impact on the genetic diversity of the different regions studied.

## Figures and Tables

**Table 1:** Information on the sampling site, coordinate, saline and alkaline concentration, and sample size of *M. nipponense* in the saline–alkaline regions of China.

Sampling Site	Coordinate	Water Area	Salinity (‰)	Alkalinity (mmol)	Sample Size
JX	116°21′13″; 28°56′52″	Poyang Lake	0	0.8	35
GS	104°18′8″; 37°11′12″	Yellow River	10	2.4	35
HLJ	124°11′35″; 46°47′37″	Lianhuanhu River	2	5.1	35
JL	124°29′35″; 45°8′45″	Chaganhu River	1	3.3	35
NMG	121°38′6″; 46°6′3″	Nenjiang River	0	1.1	35
SD	117°58′38″; 37°23′17″	Coastal mudflat	3	6.5	35
TJ	117°10′34″; 38°52′27″	Coastal mudflat	2	3.5	35
NX	106°24′23″; 38°25′46″	Yellow River	1	3.7	35
JS	120°51′58″; 32°49′36″	Coastal mudflat	2	2.8	35

**Table 2:** Summary statistics for D-loop polymorphisms of *M. nipponense*.

Sampling Sites	Number of Sizes	Number of SNPs	Number of Haplotypes (h)	Haplotype Diversity (*h*)	Nucleotide Diversity (π)
JX	32	125	18	0.879	0.031
GS	30	102	20	0.954	0.015
HLJ	33	74	31	0.996	0.016
JL	30	129	29	0.998	0.034
NMG	26	58	22	0.982	0.013
SD	20	73	19	0.995	0.012
TJ	32	87	25	0.972	0.032
NX	31	142	28	0.987	0.037
JS	30	145	30	1	0.032
Total	264	231	89	0.941	0.032

**Table 3:** Analysis of molecular variance (AMOVA) results for the *M. nipponense*.

Source of Variation	*df*	Sum of Squares	Variance Components	Percentage Variation %
Among sites	8	3797.31	15.32 Va	36.81
Within sites	255	6705.13	26.29 Vb	63.19
Total		10,502.43	41.61	100
Fixation Index	Fst: 0.36810 (*p*-value = 0.00000)			

**Table 4:** Matrix of pairwise Fst values of 9 *M. nipponense* sampling sites.

	JX	GS	HLJ	JL	NMG	SD	TJ	NX	JS
JX		0.000 *	0.000 *	0.000 *	0.000 *	0.000 *	0.000 *	0.000 *	0.000 *
GS	0.278		0.000 *	0.000 *	0.000 *	0.000 *	0.000 *	0.000 *	0.000 *
HLJ	0.479	0.695		0.000 *	0.000 *	0.423	0.000 *	0.000 *	0.000 *
JL	0.215	0.430	0.214		0.000 *	0.000 *	0.000 *	0.000 *	0.000 *
NMG	0.523	0.745	0.118	0.293		0.000 *	0.000 *	0.000 *	0.000 *
SD	0.328	0.000	0.750	0.477	0.812		0.02703 *	0.000 *	0.000 *
TJ	0.183	0.412	0.382	0.077	0.441	0.465		0.000 *	0.000 *
NX	0.090	0.384	0.484	0.244	0.518	0.422	0.218		0.000 *
JS	0.121	0.269	0.467	0.198	0.501	0.303	0.142	0.175	

Pairwise Fst, (below the diagonal); significance of corresponding *p*-values (above the diagonal) based on pairwise differences in the concatenated mtDNA sequences. *: *p* < 0.05 as per false discovery rate correction [31].

**Table 5:** Genetic distance of 9 *M. nipponense* sampling sites.

	JX	GS	HLJ	JL	NMG	SD	TJ	NX	JS
JX									
GS	0.034								
HLJ	0.058	0.055							
JL	0.051	0.046	0.041						
NMG	0.064	0.061	0.025	0.044					
SD	0.034	0.020	0.055	0.046	0.061				
TJ	0.046	0.042	0.047	0.044	0.051	0.042			
NX	0.047	0.049	0.065	0.060	0.071	0.048	0.057		
JS	0.048	0.041	0.071	0.060	0.078	0.040	0.053	0.061	

## Data Availability

The data presented in this study are available on request from the corresponding authors.

## Supplementary Materials

The following supporting information can be downloaded at https://www.mdpi.com/article/10.3390/ani15020158/s1, Table S1: Haplotype distribution of *Macrobrachium nipponense* in the saline–alkaline regions, Table S2: Nucleotide diversity, alkalinity, and salinity correlation.
