# Supplementary material for: Genetic Diversity and Population Structure of Macrobrachium nipponense Populations in the Saline–Alkaline Regions of China
Source: Animals (Basel). 2025 Jan 9;15(2):158. doi: 10.3390/ani15020158 (PMC11758298; doi:10.3390/ani15020158)
Supplement: Supplementary file 1 [file animals-15-00158-s001.zip › Table S1 Haplotype distribution of Macrobrachium nipponense in the saline alkaline regions.pdf]

Supplementary data

- **Tables S1** Haplotype (rows) distribution of *M. nipponense* (columns) in the saline alkaline regions

Hap	JX	GS	HLJ	JL	NMG	SD	TJ	NX	JS	Total
H1	2									2
H2	1									1
H3		2								2
H4			7	4	20	1				32
H5			6	3						9
H6			10	5						15
H7			2	2						4
H8				1						1
H9					1					1
H10					1					1
H11					1					1
H12					1					1
H13					1					1
H14					1					1
H15			2							2
H16			1							1
H17			1							1
H18			1							1
H19							12			12
H20				1						1
H21								6		6
H22	1									1
H23								1		1
H24								2		2
H25								1		1
H26								2		2
H27								1		1
H28	1									1
H29	1									1
H30	2									2
H31								1		1
H32								1		1
H33	1									1
H34	2						5	2	4	13
H35	1									1
H36								1		1
H37							2			2
H38									1	1
H39									1	1
H40									1	1
H41									1	1
H42				1						1
H43			3							3

H44						1				1
H45		25				18			1	44
H46	12							2	1	15
H47								2		2
H48									2	2
H49								1		1
H50									1	1
H51									1	1
H52									1	1
H53									1	1
H54	1									1
H55	1									1
H56	0								1	1
H57	0						1			1
H58	3			1						4
H59								1		1
H60	1									1
H61								1		1
H62									1	1
H63		1		7			11		1	20
H64								1		1
H65									1	1
H66								1		1
H67				1						1
H68							1			1
H69				1						1
H70				1						1
H71		1								1
H72								1		1
H73								1		1
H74				1						1
H75									1	1
H76									1	1
H77									1	1
H78									1	1
H79									1	1
H80									1	1
H81									1	1
H82									1	1
H83									1	1
H84								1		1
H85									1	1
H86	1			1						2
H87	1									1
H88								1		1
H89		1								1
Total	32	30	33	30	26	20	32	31	30	264
