# Supplementary material for: Genetic Diversity and Population Structure of Macrobrachium nipponense Populations in the Saline–Alkaline Regions of China
Source: Animals (Basel). 2025 Jan 9;15(2):158. doi: 10.3390/ani15020158 (PMC11758298; doi:10.3390/ani15020158)
Supplement: Supplementary file 1 [file animals-15-00158-s001.zip › Table S2. Nucleotide diversity, Alkalinity, and salinity Correlation of Macrobrachium nipponense.pdf]

- **Table S2.** Nucleotide diversity, Alkalinity, and salinity Correlation of *Macrobrachium nipponense* in the saline alkaline regions

	Normal distribution	Pearson correlation coefficient	Spearman correlation coefficient	P-value
π and salinity	yes	-0.4022	\	0.2832
π and alkalinity	no	\	0.03347	0.9319

π: Nucleotide diversity
